# Psychometric properties of telepressure measures in the workplace and private life among French-speaking employees

**DOI:** 10.1186/s40359-025-02616-0

**Published:** 2025-04-03

**Authors:** Raphaël Semaan, Liudmila Gamaiunova, Patricia Pereira Teixeira, Urs M. Nater, Raphaël Heinzer, José Haba-Rubio, Peter Vlerick, Ruben Cambier, Patrick Gomez

**Affiliations:** 1 Department of Occupational and Environmental Health, Unisanté, Center for Primary Care and Public Health & University of Lausanne, Lausanne, Switzerland; 2 Department of Clinical and Health Psychology, University of Vienna, Vienna, Austria; 3 University Research Platform “The Stress of Life – Processes and Mechanisms Underlying Everyday Life Stress”, University of Vienna, Vienna, Austria; 4 Department of Medicine, Center for Investigation and Research on Sleep, Lausanne University Hospital (CHUV), and University of Lausanne, Lausanne, Switzerland; 5 Department of Work, Organisation and Society, Ghent University, Ghent, Belgium

**Keywords:** Autonomy paradox, Confirmatory factor analysis, Digital wellbeing, Information and communication technology, Health, Private life telepressure, Psychological detachment from work, Stress, Workplace telepressure

## Abstract

**Background:**

Workplace telepressure and private life telepressure refer to the preoccupation with and the urge to respond quickly to electronic messages from people at work or in private life, respectively. We aimed to adapt and validate workplace and private life telepressure measures in French and to explore their nomological networks and relationships with psychological health and wellbeing.

**Methods:**

Participants were recruited via flyers, local press, and social media to complete two online surveys. Participants had to be French-speaking employees working in Switzerland and regularly using information and communication technologies for work purposes. The sample included 347 employees (200 females, 146 males, one nonbinary individual; mean age: 36.8 years) who completed both surveys. The first questionnaire assessed sociodemographic characteristics and the workplace and private life telepressure measures. The second questionnaire, which was administered approximately two weeks later, assessed complementary sociodemographic characteristics, nomologicals (five technostress creators, workaholism, neuroticism, conscientiousness, and mindfulness), measures of psychological health and wellbeing (depression, anxiety, stress, and psychological detachment from work), and the two telepressure measures.

**Results:**

Both telepressure measures exhibited strong psychometric properties, including validity, reliability, and measurement invariance across age, gender, and time. Confirmatory factor analysis revealed that the two-factor model (preoccupation and urge factors) provided a better fit than did the one-factor model for both measures. Correlation analyses revealed that both telepressure measures were significantly positively associated with techno-invasion, techno-complexity, techno-insecurity, workaholism, and neuroticism and negatively associated with mindfulness. However, only workplace telepressure was significantly associated with techno-overload, and neither telepressure measure was significantly associated with techno-uncertainty. Structural equation modeling showed that workplace telepressure significantly predicted stress, anxiety, depression, and psychological detachment from work, whereas private life telepressure significantly predicted stress, anxiety, and psychological detachment from work, but not depression. Most effects were significantly greater for workplace telepressure than for private life telepressure.

**Conclusions:**

This study confirms the validity of the workplace telepressure and private life telepressure measures for use in French-speaking populations and contributes to our understanding of the role of these two constructs in employees' psychological health and wellbeing.

**Supplementary Information:**

The online version contains supplementary material available at 10.1186/s40359-025-02616-0.

## Background

Modern information and communication technology (ICT) devices such as computers, laptops, and smartphones are important tools in the daily working life of many employees [1, 2]. Asynchronous ICT-mediated communication (e.g., emails) allows employees flexibility and control in choosing when and where to handle received messages. However, several surveys and studies (e.g., [3, 4]) have revealed that many employees have limited or no response flexibility and feel the need to be continuously connected to the workplace and to respond promptly to incoming work-related ICT messages during work hours and off-job time. This phenomenon is called the autonomy paradox [5–7]. To better characterize the ambivalence of employees’ relationships with ICTs and mobile technology, Vanden Abeele proposed a new definition of digital wellbeing, describing it as “a subjective individual experience of optimal balance between the benefits and drawbacks obtained from mobile connectivity” (p. 938) [8]. One possible challenge in achieving and maintaining digital wellbeing is workplace telepressure.

### The concept and measurement of workplace telepressure

The concept of workplace telepressure was first introduced by Barber and Santuzzi in 2015, who defined it as “the combination of a strong urge to be responsive to people at work through message-based ICTs with a preoccupation with quick response times” (p. 172) [9]. Workplace telepressure is a psychological state that can be experienced when employees begin to view asynchronous communication technologies as similar to synchronous communication (e.g., face-to-face communication), which generally require immediate responses [9]. According to self-determination theory [10], the motivation to stay connected via ICT use can be either controlled (e.g., feeling bad for not using ICT) or autonomous (e.g., recognizing the importance of staying connected to achieve personal goals) [10, 11]. Hu and colleagues categorized the experience of workplace telepressure as a controlled motivational factor in their taxonomy of ICT-related constructs [7].

In their seminal work, Barber and Santuzzi presented the development and validation of a scale to measure workplace telepressure in English-speaking employees [9]. Starting with a pool of eight items, the scale was tested for one- and two-factor models. In line with the conceptualization of the construct, their final revised 6-item version showed that workplace telepressure had two highly correlated factors, i.e., preoccupation and urge, each with three items. The authors further stated that workplace telepressure can preferably be used as a one-factor model because of the high inter-factor correlation in the two-factor model and the risk of deficiency when a subset of items is used. The reliability of the original scale has been assessed in several studies, with the results consistently demonstrating adequate to excellent internal consistency [12–15].

Since the conceptualization of workplace telepressure, the original English measure has been translated into several languages. For example, Pfaffinger et al., Cianci et al., Reimann et al., and Zinke et al. all used a German version in their research [16–19]. The workplace telepressure measure also exists in Dutch [20], Arabic [21], Russian [22], and Chinese [23]. Dose et al. developed but did not validate a French version [24]. Considering the relevance of workplace telepressure in studying the effects of ICTs on psychological functioning, health, and wellbeing, there is a need for a validated workplace telepressure measure in French.

### Workplace telepressure’s nomological network

Workplace telepressure joins a pool of other constructs, tapping into the spheres of the work environment and psychological functioning. These constructs form a nomological network [25], which represents their observable manifestations and interrelations. Theoretical and empirical linkages of workplace telepressure with other constructs allow for the establishment of its validity. The following constructs are considered possible workplace telepressure nomologicals.

Technostress creators refer to various factors contributing to the stress experience induced by using ICTs in organizations. These factors can be organized into five dimensions [26]: techno-overload, i.e., situations where employees feel that ICTs force them to work faster and longer; techno-invasion, i.e., situations where employees feel that their private life is being invaded by work ICTs; techno-complexity, i.e., situations where the use of ICTs leads to the feeling of inadequacy in relation to technological skills and leads to time expenditure and effort in learning the necessary skills; techno-insecurity, i.e., situations where employees are unsure about keeping their job due to ICT automatization or more skillful coworkers; and techno-uncertainty, i.e., situations where ICT upgrades and changes create the necessity of constant learning and education. Techno-invasion has conceptual similarities with workplace telepressure. In fact, employees who face higher ICT demands related to response expectations and constant availability, a core component of invasion, are more likely to experience workplace telepressure [9]. Similarly, techno-overload and workplace telepressure share conceptual similarities, as both stem from ICT-related demands that exceed general work overload (e.g., constant availability, rapid responses, and technological malfunctions). Workplace telepressure specifically arises from message-based ICT demands, making it more closely associated with perceptions of technological overload than with general work overload [9, 27]. A positive correlation between workplace telepressure and techno-overload has been found by Barber and Santuzzi: r = 0.31 [9]. To our knowledge, no studies have examined the associations between workplace telepressure and the other three technostress creators.

Workaholism is conceptualized as a negative psychological state that entails excessive work due to an irresistible internal drive to engage in professional activities [28]. This compulsive and excessive attitude toward work is associated with several negative outcomes, including burnout and health complaints [29]. Considering that workaholism includes an attitude of compulsiveness related to professional aspects of life, its association with workplace telepressure is plausible. Accordingly, significant positive relationships between the two constructs ranging from r = 0.19 to r = 0.35 have been reported [9, 30–32].

Personality traits are psychological characteristics that are quite stable over time and can partly explain an individual’s behavior [33]. Neuroticism and conscientiousness influence feelings of pressure in the work environment. Neuroticism is associated with tendencies such as anxiety, impulsiveness, and worry [34]. Consequently, employees exhibiting high levels of neuroticism may be prone to feelings of urge and preoccupation when confronted with work-related emails or tasks from colleagues compared with employees low in neuroticism [9]. Conscientiousness is associated with efficiency, liableness, and productivity [34]. Conscientious employees may feel compelled by a drive for productivity and efficiency upon receiving work-related messages from colleagues. This drive may lead them to feel a heightened urge for and preoccupation with promptly responding to these messages. Indeed, significant positive relationships between workplace telepressure and neuroticism, on the one hand, and conscientiousness, on the other hand, have been reported, with r = 0.19 [30, 31], and r = 0.11 [9], respectively. However, others have not found significant associations between workplace telepressure and conscientiousness [9, 30, 31].

Mindfulness can be defined as a state of mind characterized by being attentive and aware of what is happening at the moment [35]. People vary in their propensity and willingness to maintain an attentive and aware stance, with different personal factors and training in mindfulness playing important roles. The attentive awareness of one’s own cognitive, affective, and behavioral tendencies leads to a reduction in automatic processes [36], a decrease in urge [37], and enhanced self-regulation [38]. Mindfulness has been found to be associated with several benefits in the workplace, including increased psychological detachment from work [39], a reduction in burnout [40], and the mitigation of the negative role of technostress creators [41]. Considering that the construct of workplace telepressure comprises the dimensions of urge and preoccupation, it can be hypothesized that it is negatively associated with mindfulness. In line with this hypothesis, significant and negative correlations between the two constructs ranging from r = −0.37 to r = −0.46 have been reported [42–44].

### Workplace telepressure’s relationship with psychological health and wellbeing

Workplace telepressure can be expected to lead to negative subjective wellbeing and health outcomes. In the framework of the job demands-resources (JD-R) model [45], job demands can be defined as multifaceted aspects of the professional environment that create conditions for substantial cognitive and emotional effort. Workplace telepressure, comprising the urge to respond to and the preoccupation associated with work-related messages, can be seen as a reaction to the job demands introduced by the use of ICT and consequently as a contributor to the experience of controlled motivation [7]. Thus, the state of workplace telepressure might motivate workers’ continued connection to professional activities, both during and beyond working hours [9]. The chronic state of effort induced by constant connection to work and the inability to restore depleted resources may subsequently lead to exhaustion and impaired health [46]. Considering that workplace telepressure can contribute to the depletion of physical and mental resources and reduce the potential for recovery, it can be hypothesized that it could negatively affect psychological health and lead to increases in stress, anxiety, and depression. Previous research provides initial support for this hypothesis: workplace telepressure has been found to be significantly positively associated with stress, with the strength of the associations ranging from r = 0.12 to r = 0.51 [16, 18, 20, 47]; anxiety, with correlations between r = 0.15 and r = 0.35 [43, 48]; and depressive symptoms, with a correlation of r = 0.38 [43]. While the evidence of the association between depression and workplace telepressure is still limited, a related construct, excessive use of ICTs, has been consistently linked to depression [49–51].

Workplace telepressure might also affect indices of wellbeing, such as psychological detachment from work. This construct refers to mental disengagement from professional concerns and manifests in ceasing work-related tasks and abstaining from thinking about work during off-hours [52, 53]. Psychological detachment from work is an essential condition for recovery from work [54]. The preoccupation and urge related to work messages can potentially disrupt disengagement from work. A significant and negative association between workplace telepressure and psychological detachment from work has been reported in several studies, with associations ranging from r = −0.20 to β = −0.40 [15, 18, 32, 55].

### Private life telepressure

The preoccupation with and the urge to respond to messages are also relevant for private life technology-based interactions [12]. Cambier et al. proposed a distinct construct of private life telepressure, i.e., the preoccupation with and urge to respond quickly to personal ICT messages [56]. A Dutch measure allowing the differentiation of the telepressure source (private or work sphere) was introduced [57], and a confirmatory factor analysis supported a model with four first-order (preoccupation and urge) and two second-order factors (workplace telepressure and private life telepressure). Measurement invariance across age was also shown. A positive correlation between the second-order factors (young adulthood sample: r = 0.30, middle adulthood sample: r = 0.59) suggests that workplace telepressure and private life telepressure are related but nevertheless distinct. The internal consistency of the measures ranged from very good to excellent.

There is limited knowledge about private life telepressure’s nomological network and relationships with measures of psychological health and wellbeing. In contrast to research on workplace telepressure, no studies have investigated the link between private life telepressure and technostress creators, workaholism, or mindfulness. Private life telepressure during work hours has been found to be significantly negatively associated with conscientiousness (r = −0.14) and significantly positively associated with neuroticism (r = 0.23) [56], stress (r = 0.39), and affective rumination toward work issues during leisure time (a construct that shares strong similarities with psychological detachment from work; r = 0.44) [47]. These findings, albeit limited, suggest that private life telepressure represents an important construct for studying the effects of ICTs on behavior, health, and wellbeing. Thus, further exploration of this construct and validation of a corresponding measure represent valuable contributions to the research domain.

### This study

This study focuses on the adaptation and validation of the French version of the workplace telepressure measure and the exploration of the related construct of private life telepressure. First, we aim to confirm the two-factor structure of the French version of the workplace telepressure measure and test its measurement invariance, reliability, and validity. Second, we aim to explore the construct of private life telepressure and to validate the French version of its measure with an assessment of its factor structure, reliability, and relationship with other constructs, thus shedding light on its implications for psychological health and wellbeing. We have three sets of hypotheses for both workplace telepressure (H1 to H3) and private life telepressure (H4 to H6). These hypotheses are based on the literature reviewed above. We hypothesize that the French version of the workplace telepressure measure will:

- H1. Have (a) a two-factor structure (preoccupation and urge factors), (b) measurement invariance across age, gender, and time, and (c) high reliability.
- H2. Demonstrate convergent and discriminant validity and show significant and positive associations with (a) techno-overload, (b) techno-invasion, (c) workaholism, and (d) neuroticism and a significant and negative association with (e) mindfulness. The questions about the associations between workplace telepressure and techno-complexity, techno-insecurity, techno-uncertainty, and conscientiousness are treated as exploratory issues.
- H3. Demonstrate criterion (predictive) validity and show significant and positive associations with (a) stress, (b) anxiety, and (c) depression and a significant and negative association with (d) psychological detachment from work.

We hypothesize that the French version of the private life telepressure measure will:

- H4. Have (a) a two-factor structure (preoccupation and urge factors), (b) measurement invariance across age, gender, and time, and (c) high reliability.
- H5. Demonstrate convergent and discriminant validity and show a significant and positive association with (a) neuroticism and a significant and negative association with (b) conscientiousness. The questions about the associations between private life telepressure and technostress creators, workaholism, and mindfulness are treated as exploratory issues.
- H6. Demonstrate criterion (predictive) validity and show a significant and positive association with (a) stress and a significant and negative association with (b) psychological detachment from work. The questions about the associations between private life telepressure and anxiety and depression are treated as exploratory issues.

## Methods

### Participants

Participants were recruited via flyers, the local press, and social media platforms. To take part in the study, participants had to be French-speaking employees aged 18 and older who work in Switzerland and regularly use ICT for work purposes. They were asked to respond to two online surveys with an interval of at least one week. Among the 403 participants who completed the first questionnaire, 56 did not complete the second questionnaire. Thus, a final sample of 347 participants was retained for data analysis. The sample consisted of 200 female participants (57.6%), 146 male participants (42.1%), and one nonbinary participant (0.3%). The mean age of the sample was 36.8 years. Participants had been in their current jobs for an average of 5.1 years and worked an average of 39.1 h per week. Participants were employed at an average of 91.7% of a full-time position. Table S1 in the supplementary material (see Additional file 1) provides additional details. Participants received 15 Swiss francs for completing both questionnaires.

Our sample size was determined to ensure adequate power for detecting small but meaningful correlations in the range of |r|= 0.15 to 0.20 at α = 0.05 with 80% power. This decision was guided by both theoretical and empirical considerations. Theoretically, correlations of this magnitude are meaningful in workplace psychology [58, 59], as they can reflect important but subtle relationships between workplace telepressure and psychological outcomes. Empirically, prior research indicates that associations between workplace telepressure and related constructs often fall within this range (e.g., [9, 30, 47, 60]). Standard power calculations based on Cohen [61] indicate that detecting correlations of |r|= 0.15 to 0.20 requires a sample size between *N* = 193 and *N* = 346. Our final sample of *N* = 347 aligns with these recommendations, ensuring sufficient power for our primary analyses while necessitating cautious interpretation of smaller effects.

### Procedure

The study consisted of an entry questionnaire (Time 1), a second questionnaire (Time 2), and an ambulatory assessment [62]. The ambulatory assessment is not relevant to the present study.

Individuals who contacted the research team received an email with the study information sheet and a link to the entry questionnaire. This questionnaire assessed inclusion and exclusion criteria for the ambulatory part of the study and included sociodemographic, health-related, and the workplace telepressure and private life telepressure measures. Participants had to tick a box to consent to their data being saved and processed. At least one week later (M = 14.9, SD = 7.5 days), participants were invited to complete the second questionnaire assessing complementary sociodemographic and health-related characteristics, psychological constructs, and for a second time workplace telepressure and private life telepressure. The study was conducted in French.

### Measures

#### Workplace telepressure and private life telepressure

Workplace telepressure and private life telepressure were measured with French versions based on Barber and Santuzzi’s workplace telepressure measure and its later adaptation to the private life domain by Cambier et al. [9, 56]. Both scales consisted of six items. To enhance contextual clarity and in line with Cambier [57], we included the terms "work-related" and “personal” in five items of the workplace telepressure and private life telepressure measures, respectively. Before implementation, two bilingual members of the research team independently translated both Barber and Santuzzi’s original workplace telepressure measure and Cambier et al.’s adapted private life telepressure measure from English to French with assistance from a member of the Language Center of the University of Lausanne, Lausanne, Switzerland, who provided her own French version. The research team then reviewed and resolved the discrepancies between the two French translations. The obtained version was filled in by 78 colleagues, and their feedback was used to make a final minor change.

Participants were instructed to reflect on how they use technology to communicate with people in their professional [personal] environment, focusing on messaging technologies related to work [to their personal life] that allow them to control when they respond (email, text messages, voicemail, etc.). A sample item reads as follows: “I can’t stop thinking about a work-related [personal] message until I’ve responded”). The items are scored on a 5-point Likert scale (1 = “Strongly disagree” to 5 = “Strongly agree”), with a higher score representing a higher level of workplace or private life telepressure. The complete set of items is available in both French and English in the supplementary material (see Additional file 1).

#### Technostress creators

Technostress creators were assessed with the technostress creators scale consisting of five subscales [26, 63]: techno-overload (4 items), techno-invasion (4 items), techno-complexity (4 items), techno-insecurity (5 items), and techno-uncertainty (4 items). Sample items for the subscales read as follows: techno-overload: “I am forced by this technology to work much faster”; techno-invasion: “I feel my personal life is being invaded by this technology”; “techno-complexity: “I do not know enough about this technology to handle my job satisfactorily”; techno-insecurity: “I have to constantly update my skills to avoid being replaced”; techno-uncertainty: “There are constant changes in computer software in our organization”. The items are scored on a 7-point Likert scale (1 = “Strongly disagree” to 7 = “Strongly agree”), with a higher score indicating more technostress creators. The reliability indices were as follows: techno-overload (α = 0.86, ω = 0.87), techno-invasion (α = 0.80, ω = 0.81), techno-complexity (α = 0.87, ω = 0.88), techno-insecurity (α = 0.79, ω = 0.82), and techno-uncertainty (α = 0.92, ω = 0.92).

#### Workaholism

Workaholism was measured with the 10-item Dutch Work Addiction Scale [28, 64], with a sample item reading as follows: “I often feel that there’s something inside me that drives me to work hard.” The items are scored on a 4-point Likert scale (1 = “Never” to 4 = “Always”), with higher scores indicating higher levels of workaholism. The reliability indices were as follows: α = 0.85, ω = 0.85.

#### Neuroticism and conscientiousness

Neuroticism and conscientiousness were measured with 8 items and 9 items from the Big Five Inventory, respectively [65, 66]. The inventory starts with a stem “I see myself as someone who…”, followed by the items. Sample items are “Get nervous easily” (neuroticism) and “Perseveres until the task is finished” (conscientiousness). The items are scored on a 5-point Likert scale (1 = “Disagree strongly” to 5 = “Agree strongly”), with a higher score indicating greater neuroticism and conscientiousness. The reliability indices were as follows: neuroticism (α = 0.81, ω = 0.82) and conscientiousness (α = 0.79, ω = 0.77).

#### Mindfulness

To measure mindfulness, we used the 15-item Mindful Attention Awareness Scale [35, 67]. A sample item reads as follows: “It seems I am running on automatic, without much awareness of what I’m doing”. The items are scored on a 6-point Likert scale (1 = “Almost always” to 6 = “Almost never”), with a higher score indicating a higher level of mindfulness. The reliability indices were as follows: α = 0.87, ω = 0.86.

#### Depression, anxiety, and stress

Depression, anxiety, and stress were measured with the 21-item Depression, Anxiety, and Stress Scale (DASS) [68, 69]. Sample items for the subscales read as follows: depression: “I felt I wasn’t worth much as a person”; anxiety: “I felt I was close to panic”; and stress: “I found it difficult to relax”. The items are scored on a 4-point Likert scale (0 = “does not apply to me” to 3 = “applies to me entirely, or the vast majority of the time”), with a higher score indicating a higher level of depression, anxiety, and stress over the past week. The reliability indices were as follows: depression (α = 0.88, ω = 0.89), anxiety (α = 0.80, ω = 0.82), and stress (α = 0.83, ω = 0.84).

#### Psychological detachment from work

Psychological detachment from work was measured with the 4-item psychological detachment subscale of the recovery experience questionnaire [52, 70], with a sample item reading as follows: “I distance myself from my work”. The items are scored on a 5-point Likert scale (1 = “I do not agree at all” to 5 = “I fully agree”), with higher scores indicating a greater level of psychological detachment from work. The reliability indices were as follows: α = 0.91, ω = 0.90.

### Analytical approach

All analyses were performed using R version 4.3.0 (R Core Team, 2023) [71]. To test the one-factor and two-factor models of the French workplace telepressure and private life telepressure measures, confirmatory factor analysis (CFA) was performed using the lavaan package (v.0.6.16) [72]. Owing to data nonnormality, we used maximum likelihood estimation with robust standard errors and a Satorra-Bentler scaled test statistic. Model fit was evaluated using the Tucker–Lewis index (TLI) [73], the comparative fit index (CFI) [74], the root-mean-square error of approximation (RMSEA) [75], the standardized root-mean-square residual (SRMR) [76], and the chi-square goodness-of-fit test. We used the recommended thresholds for indices of close fit, i.e., TLI ≥ 0.90 [77], CFI ≥ 0.95 [73], RMSEA ≤ 0.08 [78], and SRMR ≤ 0.08 [79].

Measurement invariance of the French measures was assessed for gender (males and females), age (younger and older, split at 34 years), and measurement time (Time 1 and Time 2). The age split at 34 years corresponds to the median age of our sample, ensuring an approximately equal distribution of younger and older participants for measurement invariance testing. We followed the recommended steps for establishing configural, metric, and scalar invariance [80]. At each step, the model fits were compared to ensure that the model was not significantly worse than the previous model (e.g., configural invariance model vs. metric invariance model). To establish invariance at each step, we relied on the following criteria suggested in the literature [81]: < 0.010 change in CFI, < 0.015 change in RMSEA, and < 0.030 change in SRMR (metric invariance) or < 0.015 change in SRMR (scalar invariance).

Reliability indices — Cronbach’s α and McDonald’s ω [82, 83] were computed using the semTools package (v.0.5.6.) [84]. McDonald’s ω is a more robust reliability index that is based on a factor analytic approach and does not require tau equivalence [85]. Following DeVellis [86], we interpret Cronbach’s α and McDonald’s ω greater than 0.7, 0.8, and 0.9 as “respectable”, “very good”, and “excellent”, respectively. Test–retest reliability was assessed as a Pearson’s correlation coefficient between the scores of the workplace telepressure and private life telepressure measures at Time 1 and Time 2.

Convergent validity was assessed using Fornell and Larcker’s criterion,^83^ which consists of estimating the average variance extracted (AVE), i.e., the amount of variance in the items that can be explained by a latent variable. With a latent construct explaining more than 50% of the item variance (AVE > 0.50), convergent validity can be established. AVE values were computed with the semTools package (v.0.5.6.) [84]. Discriminant validity was assessed (1) by estimating whether the AVE for each construct was greater than the squared correlation of the construct with other constructs [87] and (2) by estimating the heterotrait–monotrait (HTMT) ratio of correlation using the recommended threshold of 0.85, with values closer to 1.00 indicating a lack of discriminant validity [88].

Associations among variables in the nomological network (with workplace telepressure and private life telepressure measured at Time 1 and the other variables measured at Time 2) were assessed via the calculation of Pearson’s correlation coefficients and computed with the package apaTables (v.2.0.8) [89]. Criterion (predictive) validity was tested via structural equation modeling, with workplace telepressure and private life telepressure measured at Time 1 and subjective health and wellbeing variables measured at Time 2. Fit of the measurement model (with the same indices as outlined above for the CFA) and structural model estimates were assessed with the lavaan package (v.0.6.16) [72]. The significance level for all tests was set at *p *< 0.05.

The nonbinary participant was included in all analyses except in gender invariance analyses due to the small sample size of the nonbinary group.

## Results

### Workplace telepressure measure

#### Confirmatory factor analysis

**Fig. 1:**
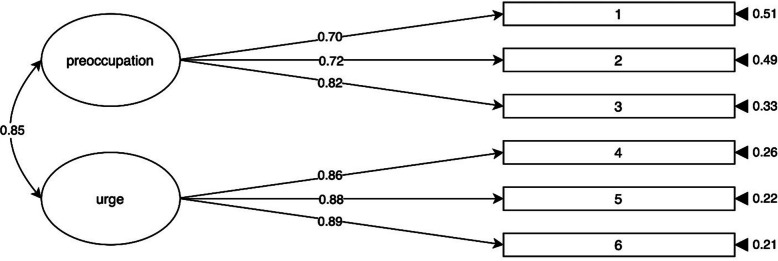
Two-factor structure of the French version of the workplace telepressure measure

The two-factor model (with preoccupation and urge factors) and the one-factor model (with a composite workplace telepressure factor) were tested with the Time 1 data (*n* = 347). The results presented in Table 1 show that the two-factor model demonstrated a better fit than the one-factor model. The graphical representation of the two-factor model in Fig. 1 shows latent variable correlations, standardized factor loadings, and standard errors.

**Table 1:** Fit indices for one-factor and two-factor models of the workplace telepressure and private life telepressure measures

Fit index	Workplace telepressure		Private life telepressure	
	**One-factor**	**Two-factor**	**One-factor**	**Two-factor**
Robust TLI	0.91	0.97	0.96	1.00
Robust CFI	0.95	0.98	0.94	1.00
Robust RMSEA	0.15	0.09	0.15	0.00
SRMR	0.05	0.03	0.04	0.01
Scaled χ2	60.83 (df = 9, *p* < 0.001)	23.47 (df = 8, *p* = 0.003)	50.57 (df = 9, *p* < 0.001)	6.44 (df = 8, *p* = 0.598)

*TLI*: Tucker‒Lewis index, *CFI*: Comparative fit index, *RMSEA*: Root mean square error of approximation, *SRMR*: Standardized root mean square residual

#### Measurement invariance

The measurement invariance tests for the workplace telepressure measure for age, gender, and time are presented in Table 2.

**Table 2:** Measurement invariance tests of the workplace telepressure and private life telepressure measures for age, gender, and time

Model type	Workplace telepressure						Private life telepressure					
	**CFI**	**RMSEA**	**SRMR**	**∆ CFI**	**∆ RMSEA**	**∆ SRMR**	**CFI**	**RMSEA**	**SRMR**	**∆ CFI**	**∆ RMSEA**	**∆ SRMR**
**Age**												
Configural	0.981	0.094	0.026				0.997	0.046	0.019			
Metric	0.982	0.081	0.034	−0.001	−0.013	0.008	0.997	0.041	0.033	0.00	−0.005	0.015
Scalar	0.984	0.071	0.035	0.001	−0.01	0.001	0.994	0.051	0.036	−0.003	0.01	0.003
**Gender**												
Configural	0.984	0.087	0.028				0.997	0.044	0.017			
Metric	0.984	0.077	0.04	0.00	−0.01	0.011	0.998	0.036	0.025	0.001	−0.008	0.008
Scalar	0.985	0.068	0.041	0.001	−0.009	0.001	0.998	0.026	0.026	0.00	−0.01	0.001
**Time**												
Configural	0.985	0.087	0.021				1	0.00	0.012			
Metric	0.986	0.076	0.022	0.001	−0.012	0.001	1	0.00	0.017	0.00	0.00	0.005
Scalar	0.984	0.074	0.025	−0.002	−0.002	0.003	1	0.00	0.019	0.00	0.00	0.003

*CFI* Comparative fit index, *RMSEA* Root mean square error of approximation, *SRMR* Standardized root mean square residual

#### Reliability

The reliability indices were α = 0.90 and ω = 0.89 at Time 1 and α = 0.92 and ω = 0.92 at Time 2. Test–retest reliability, indexed as the Pearson correlation coefficient for the workplace telepressure score measured at Time 1 (M = 3.41, SD = 1.02) and Time 2 (M = 3.40, SD = 1.08), was r = 0.83 [95% CI: 0.79, 0.86].

#### Convergent and discriminant validity

The AVE value for the composite workplace telepressure score was 0.62. The AVE value of the urge factor (0.77) was greater than the squared correlation of the two constructs (0.73), whereas the AVE value of the preoccupation factor (0.57) was not. The HTMT index was 0.84.

#### Nomological network

The associations between workplace telepressure and the other psychological constructs are presented in Table 3.

**Table 3:** Means (M), standard deviations (SD), and correlations with 95% confidence intervals of variables in the nomological network of workplace telepressure and private life telepressure

Variable	M	SD	1	2	3	4	5	6	7	8	9	10
1. WTP	3.41	1.02										
2. PTP	2.74	1.15	0.48***									
			[0.39, 0.56]									
3. Neuroticism	2.66	0.79	0.33***	0.21***								
			[0.23, 0.42]	[0.11, 0.31]								
4. Conscientiousness	4.08	0.60	0.04	−0.06	−0.18***							
			[−0.07, 0.14]	[−0.17, 0.04]	[−0.28, −0.08]							
5. Mindfulness	3.74	0.87	−0.25***	−0.12*	−0.35***	0.26***						
			[−0.34, −0.15]	[−0.22, −0.02]	[−0.44, −0.26]	[0.16, 0.35]						
6. Workaholism	2.57	0.64	0.44***	0.14*	0.24***	0.01	−0.33***					
			[0.35, 0.52]	[0.03, 0.24]	[0.14, 0.34]	[−0.10, 0.11]	[−0.42, −0.24]					
7. Techno-overload	3.76	1.57	0.20***	0.09	0.20***	0.03	−0.24***	0.47***				
			[0.10, 0.30]	[−0.02, 0.19]	[0.10, 0.30]	[−0.07, 0.14]	[−0.34, −0.14]	[0.38, 0.54]				
8. Techno-invasion	3.31	1.56	0.39***	0.22***	0.17**	−0.02	−0.26***	0.52***	0.50***			
			[0.29, 0.47]	[0.12, 0.32]	[0.06, 0.27]	[−0.12, 0.09]	[−0.36, −0.16]	[0.44, 0.59]	[0.42, 0.58]			
9. Techno-uncertainty	4.09	1.68	0.09	0.02	0.05	0.05	−0.04	0.18***	0.36***	0.29***		
			[−0.01, 0.20]	[−0.09, 0.12]	[−0.06, 0.15]	[−0.06, 0.15]	[−0.14, 0.07]	[0.08, 0.28]	[0.27, 0.45]	[0.19, 0.38]		
10. Techno-complexity	2.37	1.38	0.20***	0.17**	0.19***	-0.12*	-0.21***	0.26***	0.42***	0.39***	0.28***	
			[0.10, 0.30]	[0.06, 0.27]	[0.08, 0.29]	[-0.22, -0.01]	[-0.31, -0.11]	[0.15, 0.35]	[0.33, 0.50	[0.29, 0.47]	[0.18, 0.37]x	
11. Techno-insecurity	2.04	1.18	0.26***	0.18***	0.16**	-0.06	0.16**	0.28***	0.38***	0.50***	0.37***	0.55***
			[0.16, 0.36]	[0.07, 0.28]	[0.05, 0.26]	[-0.17, 0.04	[-0.26, -0.05]	[0.18, 0.37]	[0.28, 0.46]	[0.42, 0.57]	[0.27, 0.45]	[0.47, 0.62]

Values in square brackets indicate the 95% confidence interval for each correlation

*WTP* Workplace telepressure, *PTP* Private life telepressure, WTP and PTP were measured at Time 1, and the other constructs were measured at Time 2

*Indicates *p* < 0.05

**Indicates *p* < 0.01

***Indicates *p* < 0.001

#### Criterion validity

**Fig. 2:**
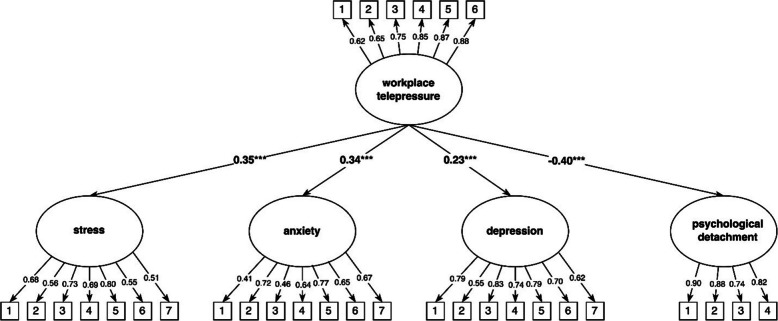
Workplace telepressure as predictor of stress, anxiety, depression, and psychological detachment from work. Notes: *** indicates *p* < 0.001

The measurement model with workplace telepressure, stress, anxiety, depression, and psychological detachment from work demonstrated a good fit: RMSEA = 0.05, TLI = 0.92, CFI = 0.93, and SRMR = 0.05. Standardized structural model estimates are presented in Table 4. As shown in Fig. 2, workplace telepressure was a significant predictor of stress, anxiety, depression, and psychological detachment from work.

**Table 4:** Standardized structural model estimates of workplace telepressure and private life telepressure (measured at Time 1) predicting stress, anxiety, depression, and psychological detachment from work (measured at Time 2)

Variable	Workplace telepressure				Private life telepressure			
	Coefficient [95% CI]	SE	Z	*p* value	Coefficient [95% CI]	SE	Z	*p* value
Stress	0.35 [0.24; 0.46]	0.06	6.27	< 0.001	0.21 [0.10; 0.33]	0.06	3.67	< 0.001
Anxiety	0.34 [0.24; 0.44]	0.05	6.73	< 0.001	0.18 [0.06; 0.30]	0.06	2.84	0.004
Depression	0.23 [0.11; 0.35]	0.06	3.67	< 0.001	0.10 [−0.01; 0.22]	0.06	1.73	0.083
Psychological detachment	−0.40 [−0.51; −0.29]	0.05	−7.34	< 0.001	−0.13 [−0.24; −0.02]	0.06	−2.25	0.024

*CI* Confidence interval, *SE* Standard error

### Private life telepressure measure

#### Confirmatory factor analysis

**Fig. 3:**
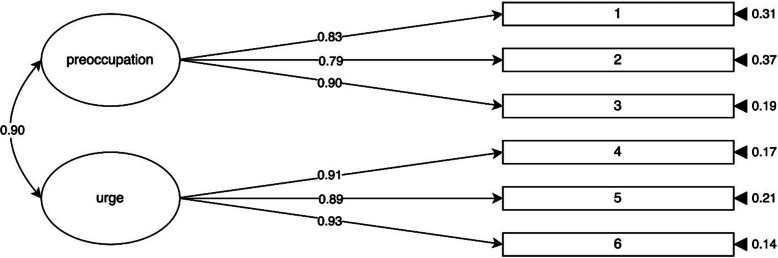
Two-factor structure of the French version of the private life telepressure measure

The two-factor model (with preoccupation and urge factors) and the one-factor model (with a composite private life telepressure factor) were tested with the data collected at Time 1 (*n* = 347). The results presented in Table 1 show that the two-factor model demonstrated a better fit than the one-factor model. The graphical representation of the two-factor model in Fig. 3 shows latent variable correlations, standardized factor loadings, and standard errors.

#### Measurement invariance

The measurement invariance tests for the private life telepressure measure for age, gender, and time are presented in Table 2.

#### Reliability

The reliability indices were α = 0.94 and ω = 0.93 at Time 1 and α = 0.93 and ω = 0.92 at Time 2. Test–retest reliability, indexed as Pearson's correlation coefficients for the private life telepressure measure measured at Time 1 (M = 2.74, SD = 1.15) and Time 2 (M = 2.73, SD = 1.12), was r = 0.74 [95% CI: 0.69, 0.78].

#### Convergent and discriminant validity

The AVE value for the composite private life telepressure score was 0.72. The AVE value of the urge factor (0.83) was greater than the squared correlation of the two constructs (0.81), whereas the AVE value of the preoccupation factor (0.70) was not. The HTMT index was 0.89.

#### Nomological network

The associations between private life telepressure and the other psychological constructs are presented in Table 3.

#### Criterion validity

**Fig. 4:**
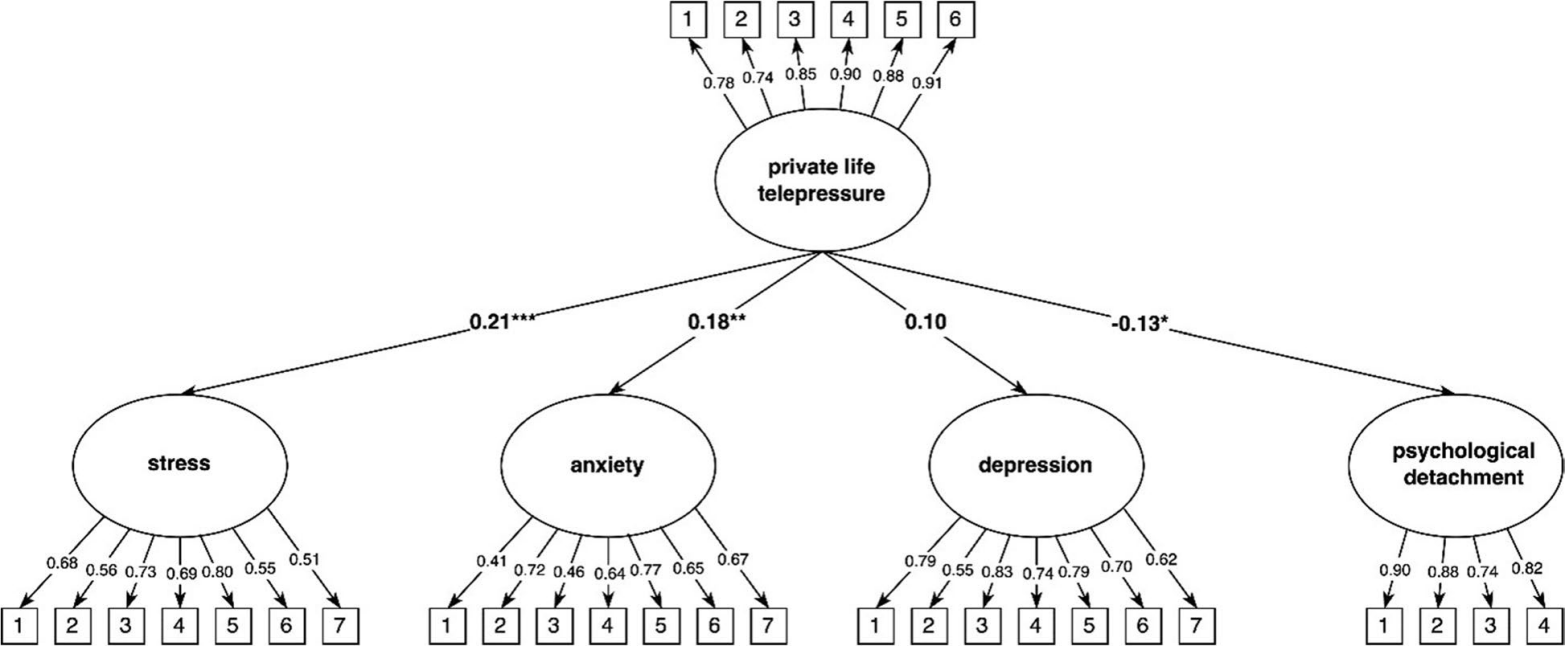
Private life telepressure as predictor of stress, anxiety, depression, and psychological detachment from work. Notes: * indicates *p* < 0.05. ** indicates *p* < 0.01. *** indicates *p* < 0.001

The measurement model with private life telepressure, stress, anxiety, depression, and psychological detachment from work demonstrated a good fit: RMSEA = 0.06, TLI = 0.92, CFI = 0.92, and SRMR = 0.06. Standardized structural model estimates are presented in Table 4. As shown in Fig. 4, private life telepressure significantly predicted stress, anxiety, and psychological detachment from work but not depression.

## Discussion

In this paper, we validated French workplace telepressure and private life telepressure measures via online surveys of a sample of 347 French-speaking employees in Switzerland.

### Workplace telepressure

The workplace telepressure measure exhibited robust psychometric properties. Testing both the two-factor model (preoccupation and urge factors) and the one-factor model (composite workplace telepressure) revealed that the two-factor model provided a superior fit. However, the high correlation between factors supports the use of a composite score, as suggested by Barber and Santuzzi [9]. Measurement invariance was confirmed across age, gender, and time, indicating the measure's suitability for diverse groups and temporal stability. These findings are in line with and extend the results of Cambier [57], who demonstrated measurement invariance across age for the Dutch workplace telepressure measure, and Hu et al., who demonstrated measurement invariance for the English workplace telepressure measure across different occupational characteristics [60]. Overall, these findings support the robustness of the workplace telepressure measure across various contexts, groups, and times.

The internal consistency of the workplace telepressure measure was very good to excellent. Similarly, the measure demonstrated high test–retest reliability. Convergent validity was supported by the value of the AVE above the recommended threshold, suggesting that items converge well together in representing the latent construct. The results indicate a partial lack of discriminant validity between the preoccupation and urge factors, as reflected by the AVE values. However, the HTMT index, below the recommended threshold (i.e., 0.85) [88], supports discriminant validity between the two factors. This suggests that while there is some overlap between the factors, they are distinguishable from one another.

As predicted, the analysis of the nomological network revealed a significant and positive association between workplace telepressure and workaholism. We anticipated a positive correlation between workplace telepressure and workaholism because workaholism is characterized by an obsessive commitment to work, often driven by irresistible internal drive rather than external demands [28, 90]. The correlation between the two constructs likely stems from the urge to stay engaged with work and the inability to disconnect, both of which are common features of workplace telepressure and workaholism. In line with our hypotheses, we also found that workplace telepressure was significantly positively correlated with techno-overload and techno-invasion. The positive association with techno-overload may arise from the urge to respond constantly to work demands. Techno-invasion can lead to persistent preoccupation with work beyond regular hours. Owing to the lack of research, we had not formulated any hypotheses regarding the associations between workplace telepressure and the other three technostress creators. We found significant and positive associations between workplace telepressure and both techno-complexity and techno-insecurity. These findings could be explained by the fact that techno-complexity introduces additional challenges, increasing the preoccupation with work-related demands and creating pressure to remain constantly engaged with work, whereas employees experiencing techno-insecurity may feel a strong urge to prove their value by staying constantly connected and responsive to work demands. Although one could speculate that techno-uncertainty may increase employees’ preoccupation with staying up-to-date, competent, and responsive to work-related messages, the correlation between workplace telepressure and techno-uncertainty was not significant. We wonder whether this could be partly due to the specific characteristics of the items used to measure techno-uncertainty, which all lack personal reference and experience (e.g., “There are constant changes in computer software in our organization”) [26], contrary to the items measuring the other four technostress creators (e.g., "I feel constant threat to my job security due to new technologies") [26]. Future studies should determine whether a scale measuring greater personal experience of techno-uncertainty would yield a significant and positive association with workplace telepressure.

As predicted, we found a significant and positive association between workplace telepressure and neuroticism, aligning with the literature showing positive associations between these two constructs [9, 30, 31]. Given the mixed findings in the literature, we did not have a hypothesis concerning the association between workplace telepressure and conscientiousness. Our findings revealed a nonsignificant and close to zero correlation between workplace telepressure and conscientiousness, in line with recent data [30, 31]. Notably, Barber and Santuzzi reported a significant but weak correlation (r = 0.11) in their first study, whereas their second study yielded a nonsignificant and very weak correlation (r = 0.07) [9]. Taken together, these results suggest that, contrary to neuroticism, conscientiousness may not be a relevant factor in understanding workplace telepressure. A few studies have investigated the associations between workplace telepressure and other personality traits (extraversion and agreeableness). These studies reported mostly nonsignificant and weak correlations [9, 30, 31].

As predicted, the analysis of the nomological network revealed a significant negative association between workplace telepressure and mindfulness, which aligns with the literature [42–44]. Together, these findings add to the literature relating mindfulness to several work-related constructs, such as workaholism [91, 92], techno-stressors [41, 93], or psychological detachment from work [94, 95]. Considering that attentive awareness is trainable and that mindfulness interventions have already been tested in the workplace environment [96], these findings call for the study of mindfulness-based approaches to prevent and reduce workplace telepressure and illustrate the importance of proper ICT-related behavioral management at large.

The predictive validity of workplace telepressure was demonstrated by its significant and small positive associations with stress, anxiety, and depression and its significant and moderate negative associations with psychological detachment from work. These findings align with research, which indicates that employees experiencing high levels of workplace telepressure have greater difficulty detaching psychologically from work [9, 18, 20, 30, 32], and report increased day-level stress [18, 20, 43], anxiety [48], and depression [43]. These findings demonstrate that workplace telepressure has the potential to negatively influence health and wellbeing. Future and ongoing studies (e.g., [62]) may provide additional evidence on the effects of workplace telepressure on physiological markers of health and test theoretically relevant mediating factors.

### Private life telepressure

One of this study’s contributions is the adaptation and validation of the French private life telepressure measure. This will allow the construct of telepressure to be explored beyond the professional domain or in boundary-crossing contexts. Like its workplace counterpart, the private life telepressure measure demonstrated the best fit for the two-factor model, with the factors preoccupation and urge being highly correlated. Thus, the recommendation of Barber and Santuzzi to use a composite measure of workplace telepressure due to a high correlation between the two factors can also be applied to the private life telepressure measure [9].

Like the workplace telepressure measure, the private life telepressure measure has sound psychometric properties. It showed excellent internal consistency, temporal stability, and invariance across age, gender, and time, allowing it to be used for group comparisons and longitudinal designs. The high value of AVE supported the measure’s convergent validity, suggesting that, on average, 72% of the variance in the construct of private life telepressure is explained by the corresponding items. The results indicate a partial lack of discriminant validity between the preoccupation and urge factors, as reflected by the AVE values. An HTMT index above the recommended threshold (i.e., 0.85 [88]) suggests poor discriminant validity between the two factors. This finding indicates that the two factors are not sufficiently distinct from one another, implying that they might measure similar or overlapping aspects of private life telepressure.

Based on work by Cambier and colleagues [56], we anticipated that private life telepressure would have a significant positive association with neuroticism and a significant negative association with conscientiousness. The exploration of the nomological network of private life telepressure demonstrated that, like workplace telepressure, it is significantly positively associated with neuroticism. In contrast, private life telepressure showed a nonsignificant and very small negative association with conscientiousness, similar in size to the association reported by Cambier et al. [56]. As observed for workplace telepressure, these findings suggest that conscientiousness might not be a relevant component within the nomological network of private life telepressure. Future studies may investigate the associations between private life telepressure and other personality traits.

Owing to a lack of research, questions relating to the associations between private life telepressure and technostress creators, workaholism, and mindfulness were treated as exploratory issues. We found that private life telepressure was significantly positively associated with techno-invasion, techno-complexity, techno-insecurity, and workaholism. These associations were weaker than those observed with workplace telepressure. This makes sense considering that these constructs concern the professional sphere.

Private life telepressure was significantly negatively associated with mindfulness, suggesting that individual differences in the level of attention to and awareness of the present moment are related to the level of telepressure not only in the professional domain but also in the private domain. These findings support the evidence from previous studies, showing a negative relationship between mindfulness and urges related to social media use [97] or a negative association between mindfulness and a compulsive pattern of use and preoccupation with a smartphone [98]. Notably, the magnitude of the association between private life telepressure and mindfulness was significantly weaker than that between workplace telepressure and mindfulness, suggesting that context may play an important role in the association between telepressure and the level of attentive awareness.

Private life telepressure demonstrated predictive validity by showing significant and positive associations with stress and anxiety and a significant and negative association with psychological detachment from work. These findings are in line with and extend reports by Cambier and Vlerick, who reported that private life telepressure experienced at work was significantly positively associated with both stress and affective rumination toward work issues during leisure time [47]. The coefficient between private life telepressure and depression was positive, albeit nonsignificant. These results are in line with previous reports demonstrating the negative effects of the communication load resulting from private e-mails and social media on perceived stress and anxiety [99] and the positive association between perceived cyber-based overload and stress [100]. Overall, these findings suggest that the negative effects of ICTs on health and wellbeing are not limited to the use of technology in the workplace but also expand to the private sphere. Indeed, the potential dangers of ICTs use for private purposes can lead to communication overload and contribute to impaired health or wellbeing through continuous communication vigilance, increased communication demands, and multitasking [101]. We note that the associations of private life telepressure with stress, anxiety, depression, and psychological detachment from work were all weaker than those obtained for workplace telepressure, suggesting that the telepressure associated with the professional use of ICTs may have a more profound effect on psychological health and wellbeing than the telepressure associated with the personal use of ICTs.

### Limitations and future research directions

While the private life telepressure measure demonstrated good psychometric properties, future studies should expand its nomological network, as several nomologicals in this study are work related. Moreover, the sample of the study was limited to French speakers working in Switzerland.

Future studies should assess the properties of these measures in French speakers in other countries (e.g., France, Belgium, Canada). Finally, future longitudinal studies applying longer time intervals between measurement waves (e.g., one year) are needed to detect other personal and work-related outcomes of both workplace telepressure and private life telepressure (e.g., depression, sickness absence, basic need satisfaction, work performance, and actual ICT use).

## Conclusions

The French versions of the workplace telepressure and private life telepressure measures exhibit robust psychometric properties, are positively associated with several technostress creators, workaholism, and neuroticism and negatively associated with mindfulness and predict anxiety, stress, and psychological detachment from work. The successful validation of these measures makes them suitable for use in both practice and research with French-speaking populations.

## Supplementary Information

Supplementary Material 1.

## Data Availability

The data that support the findings of this study are available from the corresponding authors upon reasonable request.

## References

[1] Day A, Cook R, Jones-Chick R, Myers V. Are your smart technologies killing it or killing you? Developing a research agenda for workplace ICT and worker wellbeing. In: Kelloway EK, Cooper C, editors. A research agenda for workplace stress and wellbeing. Cheltenham: Edward Elgar Publishing Limited; 2021. p. 91–118. 10.4337/9781789905021.00014.

[2] Messenger J, Vargas OL, Gschwind L, Boehmer S, Vermeylen G, Wilkens M. Working anytime, anywhere: the effects on the world of work. Publications Office of the European Union Luxembourg Eurofound. 2017. 10.2806/372726.

[3] Americans stay connected to work on weekends, vacation, and even when out sick. Am Psychol Assoc Press Room; 2013. https://www.apa.org/news/press/releases/2013/09/connected-work. Accessed 13 May 2024.

[4] Toister J. How quickly should you respond to email? Toister Performance Solutions, Inc.; 2020. https://www.toistersolutions.com/blog/how-quickly-should-you-respond-to-email. Accessed 13 May 2024.

[5] Mazmanian M, Orlikowski WJ, Yates J. The autonomy paradox: the implications of mobile email devices for knowledge professionals. Org Sci. 2013;24(5):1337–57. 10.1287/orsc.1120.0806.

[6] Day A, Barber L, Tonet J, Landers R. Information communication technology and employee well-being: understanding the “iParadox Triad” at work. In: Landers RN, editor. The Cambridge handbook of technology and employee behavior. Cambridge: Cambridge University Press; 2019. p. 580–607. 10.1017/9781108649636.022.

[7] Hu X, Park Y, Day A, Barber LK. Time to disentangle the information and communication technology (ICT) constructs: developing a taxonomy around ICT use for occupational health research. Occup Health Sci. 2021;5(1):217–45. 10.1007/s41542-021-00085-6. 33748406 10.1007/s41542-021-00085-6 PMC7962926

[8] Vanden Abeele MM. Digital wellbeing as a dynamic construct. Comm Theory. 2021;31(4):932–55. 10.1093/ct/qtaa024.

[9] Barber LK, Santuzzi AM. Please respond ASAP: workplace telepressure and employee recovery. J Occup Health Psychol. 2015;20(2):172. 10.1037/a0038278. 25365629 10.1037/a0038278

[10] Ryan RM, Deci EL. Self-determination theory and the facilitation of intrinsic motivation, social development, and well-being. Am Psychol. 2000;55(1): 68. 10.1037/0003-066X.55.1.68. 11392867 10.1037//0003-066x.55.1.68

[11] Ohly S, Latour A. Work-related smartphone use and well-being in the evening. J Person Psychol. 2014. 10.1027/1866-5888/a000114.

[12] Barber LK, Santuzzi AM. Telepressure and college student employment: the costs of staying connected across social contexts. Stress Health. 2017;33(1):14–23. 10.1002/smi.2668. 26833698 10.1002/smi.2668

[13] Barber LK, Conlin AL, Santuzzi AM. Workplace telepressure and work–life balance outcomes: the role of work recovery experiences. Stress Health. 2019;35(3):350–62. 10.1002/smi.2864. 30882979 10.1002/smi.2864

[14] Page KJ, Nastasi A, Voyles E. Did you get that thing I sent you? Mediating effects of strain and work-family conflict on the telepressure and burnout relationship. Stress Health. 2021;37(5):928–39. 10.1002/smi.3052. 33882178 10.1002/smi.3052

[15] Gillet N, Morin AJ, Fernet C, Austin S, Huyghebaert-Zouaghi T. Telepressure and recovery experiences among remote and onsite workers. J Person Psychol. 2022. 10.1027/1866-5888/a000303.

[16] Pfaffinger KF, Reif JA, Spieß E. When and why telepressure and technostress creators impair employee well-being. Int J Occup Saf Ergon. 2022;28(2):958–73. 10.1080/10803548.2020.1846376. 33164707 10.1080/10803548.2020.1846376

[17] Cianci J, Weibel D, Elfering A. Measuring work demands and resources of digitalisation: the ICT resources and stressors scale. Swiss Psychol Open. 2024;4(1):1–26. 10.48350/198511.

[18] Reimann L-E, Binnewies C, Ozimek P, Loose S. I do not want to miss a thing! consequences of employees’ workplace fear of missing out for ICT use, well-being, and recovery experiences. Behav Sci. 2024;14(1): 8. 10.3390/bs14010008. 10.3390/bs14010008 PMC10812831 38247660

[19] Zinke J, Vahle-Hinz T, Hoppe A. A longitudinal study on ICT workload in the extended stressor-detachment model: testing moderated mediation models for extended work availability and workplace telepressure. Work Stress. 2024;38(1):73–89. 10.1080/02678373.2023.2239179.

[20] Cambier R, Derks D, Vlerick P. Detachment from work: a diary study on telepressure, smartphone use and empathy. Psychol Belg. 2019;59(1):227. 10.5334/pb.477. 31328018 10.5334/pb.477 PMC6625542

[21] أبوسيف, ر. أثـر ضغط التواصل مع العمل عن بُعد على الرفاهية النفسيَّة لأعضاء هيئة التدريس بجامعة الأزهر من خلال الدور الوسيط للصلابة النفسيَّة المجلة العربية للإدارة 2022;42(4):359–380. 10.21608/aja.2022.273281. Arabic. [English translation] The impact of the pressure of staying connected to work during telework on the psychological wellbeing of faculty members at Al-Azhar University through the mediating role of psychological resilience.

[22] Shakhova Z. Deconnection strategies–a solution to decrease workplace telepressure and increase psychological detachment. [Dissertation]. The Netherlands: University of Tilburg; 2022.

[23] Kao K-Y, Chi N-W, Thomas CL, Lee H-T, Wang Y-F. Linking ICT availability demands to burnout and work-family conflict: the roles of workplace telepressure and dispositional self-regulation. J Psychol. 2020;154(5):325–45. 10.1080/00223980.2020.1745137. 32281919 10.1080/00223980.2020.1745137

[24] Dose É, Desrumaux P, Rekik M. Télépression au travail, relation entre l’utilisation de la messagerie électronique au travail et les échanges leader-membres : rôles de la reconnaissance et de la charge de travail. Travail Hum. 2019;82(2):151–81. 10.3917/th.822.0151. French. [English translation] Workplace telepressure, the relationship between the use of email at work and leader-member exchanges: roles of recognition and workload.

[25] Cronbach LJ, Meehl PE. Construct validity in psychological tests. Psychol Bull. 1955;52:281–302. 10.1037/h0040957. 13245896 10.1037/h0040957

[26] Ragu-Nathan T, Tarafdar M, Ragu-Nathan BS, Tu Q. The consequences of technostress for end users in organizations: conceptual development and empirical validation. Inf Sys Res. 2008;19(4):417–33. 10.1287/isre.1070.0165.

[27] Day A, Paquet S, Scott N, Hambley L. Perceived information and communication technology (ICT) demands on employee outcomes: the moderating effect of organizational ICT support. J Occup Health Psychol. 2012;17(4):473. 10.1037/a0029837. 23066697 10.1037/a0029837

[28] Del Líbano M, Llorens S, Salanova M, Schaufeli W. Validity of a brief workaholism scale. Psicothema. 2010;22(1):143–50. 20100441

[29] Schaufeli WB, Taris TW, Bakker AB. It takes two to tango: workaholism is working excessively and working compulsively. In: Burke RJ, Cooper CL, editors. The long work hours culture: causes, consequences and choices. Bingley: Emerald Group Publishing Limited; 2008. p. 203–26.

[30] Grawitch MJ, Werth PM, Palmer SN, Erb KR, Lavigne KN. Self-imposed pressure or organizational norms? Further examination of the construct of workplace telepressure. Stress Health. 2018;34(2):306–19. 10.1002/smi.2792. 29235229 10.1002/smi.2792

[31] Hong J, Jex S. The conditions of successful telework: exploring the role of telepressure. Int J Environ Res Public Health. 2022;19(17): 10634. 10.3390/ijerph191710634. 36078350 10.3390/ijerph191710634 PMC9517852

[32] Barber LK, Leslie S, Samaniego A. Workplace telepressure and work rumination: evidence of incremental validity beyond workaholism. Occup Health Sci. 2024:1–17. 10.1007/s41542-024-00187-x.

[33] Mount MK, Barrick MR, Scullen SM, Rounds J. Higher-order dimensions of the big five personality traits and the big six vocational interest types. Person Psychol. 2005;58(2):447–78. 10.1111/j.1744-6570.2005.00468.x.

[34] McCrae RR, John OP. An introduction to the five-factor model and its applications. J Person. 1992;60(2):175–215. 10.1111/j.1467-6494.1992.tb00970.x. 1635039 10.1111/j.1467-6494.1992.tb00970.x

[35] Brown KW, Ryan RM. The benefits of being present: mindfulness and its role in psychological well-being. J Person Soc Psychol. 2003;84(4): 822. 10.1037/0022-3514.84.4.822. 10.1037/0022-3514.84.4.822 12703651

[36] Ostafin BD. Taming the wild elephant: mindfulness and its role in overcoming automatic mental processes. In: Ostafin BD, Robinson MD, Meier BP, editors. Handbook of mindfulness and self-regulation. New York: Springer; 2015. p. 47–63. 10.1007/978-1-4939-2263-5_5.

[37] Tapper K. Mindfulness and craving: effects and mechanisms. Clin Psychol Rev. 2018;59:101–17. 10.1016/j.cpr.2017.11.003. 29169665 10.1016/j.cpr.2017.11.003

[38] Leyland A, Rowse G, Emerson L-M. Experimental effects of mindfulness inductions on self-regulation: systematic review and meta-analysis. Emot. 2019;19(1):108. 10.1037/emo0000425. 10.1037/emo0000425 29578742

[39] Mellner C, Osika W, Niemi M. Mindfulness practice improves managers’ job demands-resources, psychological detachment, work-nonwork boundary control, and work-life balance–a randomized controlled trial. Int J Workplace Health Manag. 2022;15(4):493–514. 10.1108/IJWHM-07-2021-0146.

[40] Luken M, Sammons A. Systematic review of mindfulness practice for reducing job burnout. Am J Occup Ther. 2016;70(2):7002250020p1–10. 10.5014/ajot.2016.016956. 26943107 10.5014/ajot.2016.016956 PMC4776732

[41] Ioannou A, Lycett M, Marshan A. The role of mindfulness in mitigating the negative consequences of technostress. Inf Sys Front. 2024;26(2):523–49. 10.1007/s10796-021-10239-0. 10.1007/s10796-021-10239-0 PMC8790950 35095332

[42] Thommes MS. You’ve got mail: the effect of workplace telepressure on recovery processes and the benefits of mindfulness [dissertation]. Maastricht: Maastricht University; 2015.

[43] Franzen M. ‘Inhale… Exhale… Send e-mail’_ A study examining the role of trait mindfulness on workplace telepressure and its impacts on well-being, psychological complaints and work-life balance among workers [dissertation]. Utrecht: Utrecht University; 2020.

[44] Liu B, Zhang Z, Lu Q. Influence of leader mindfulness on the emotional exhaustion of university teachers: resources crossover effect. Front Psychol. 2021;12: 597208. 10.3389/fpsyg.2021.597208. 33732180 10.3389/fpsyg.2021.597208 PMC7959755

[45] Bakker AB, Demerouti E. The job demands-resources model: state of the art. J Manag Psychol. 2007;22(3):309–28. 10.1108/02683940710733115.

[46] Demerouti E, Bakker AB. The job demands-resources model: challenges for future research. SA J Ind Psychol. 2011;37(2):01–9. 10.4102/sajip.v37i2.974.

[47] Cambier R, Vlerick P. When thoughts have no off switch: the cost of telepressure and message-based communication behaviour within boundary-crossing contexts. Occup Health Sci. 2022;6(4):545–64. 10.1007/s41542-022-00127-7. 36339884 10.1007/s41542-022-00127-7 PMC9628384

[48] He X, Gao Q, Cao Y, Bian R, Wang XH. “Always online”: how and when task interdependence and dispositional workplace anxiety affect workplace telepressure after hours. PsyCh J. 2024. 10.1002/pchj.747. 38530885 10.1002/pchj.747 PMC11317195

[49] Francis J, Kadylak T, Cotten SR, Rikard R. When it comes to depression, ICT use matters: a longitudinal analysis of the effect of ICT use and mattering on depression among older adults. Springer; 2016. p. 301–306. 10.1007/978-3-319-40542-1_49.

[50] Panova T, Lleras A. Avoidance or boredom: Negative mental health outcomes associated with use of information and communication technologies depend on users’ motivations. Comput Hum Behav. 2016;58:249–58. 10.1016/j.chb.2015.12.062.

[51] Thomée S, Eklöf M, Gustafsson E, Nilsson R, Hagberg M. Prevalence of perceived stress, symptoms of depression and sleep disturbances in relation to information and communication technology (ICT) use among young adults–an explorative prospective study. Comput Hum Behav. 2007;23(3):1300–21. 10.1016/j.chb.2004.12.007.

[52] Sonnentag S, Fritz C. The recovery experience questionnaire: development and validation of a measure for assessing recuperation and unwinding from work. J Occup Health Psychol. 2007;12(3): 204. 10.1037/1076-8998.12.3.204. 17638488 10.1037/1076-8998.12.3.204

[53] Sonnentag S, Cheng BH, Parker SL. Recovery from work: advancing the field toward the future. Annu Rev Org Psychol Org Behav. 2022;9(1):33–60. 10.1146/annurev-orgpsych-012420-091355.

[54] Sonnentag S. Recovery from fatigue: the role of psychological detachment. Am Psychol Assoc. 2011. 10.1037/12343-012.

[55] Santuzzi AM, Barber LK. Workplace telepressure and worker well-being: the intervening role of psychological detachment. Occup Health Sci. 2018;2(4):337–63. 10.1007/s41542-018-0022-8.

[56] Cambier R, Van Laethem M, Vlerick P. Private life telepressure and workplace cognitive failure among hospital nurses: the moderating role of mobile phone presence. J Adv Nurs. 2020;76(10):2618–26. 10.1111/jan.14496. 32803902 10.1111/jan.14496

[57] Cambier R. The itch for replying quickly: further insight into employees’ telepressure [dissertation]. Ghent: Ghent University; 2022.

[58] Gignac GE, Szodorai ET. Effect size guidelines for individual differences researchers. Pers Individ Dif. 2016;102:74–8. 10.1016/j.paid.2016.06.069.

[59] Funder DC, Ozer DJ. Evaluating effect size in psychological research: sense and nonsense. Adv Methods Pract Psychol Sci. 2019;2(2):156–68. 10.1177/2515245919847202.

[60] Hu XJ, Pawirosetiko JS, Santuzzi AM, Barber LK. Does your job shape your experience or interpretation of workplace telepressure? Exploring measurement invariance across occupational characteristics. Comput Hum Behav Reps. 2024;14: 100426. 10.1016/j.chbr.2024.100426.

[61] Cohen J. Statistical power analysis for the behavioral sciences. 2nd ed. London: Routledge; 1988.

[62] Semaan R, Nater UM, Heinzer R, et al. Does workplace telepressure get under the skin? Protocol for an ambulatory assessment study on wellbeing and health-related physiological, experiential, and behavioral concomitants of workplace telepressure. BMC Psychol. 2023;11(1):145. 10.1186/s40359-023-01123-4. 37138296 10.1186/s40359-023-01123-4 PMC10155671

[63] Fuhrer C. Diminuer le technostress pour que s’exprime la capacité d’absorption de l’utilisateur ? Rev Sci Gest. 2021;(3–4):57–71. Available from: https://www.cairn.info/revue-des-sciences-de-gestion-2021-3-page-57.htm. French. [English translation] Fuhrer C. Reducing technostress to enhance the user’s absorption capacity?

[64] Sandrin E, Gillet N. Validation d’une version française de la Dutch Work Addiction Scale (DUWAS). Psychol Trav Org. 2016;22(3):147–59. 10.1016/j.pto.2016.02.007. French. [English translation] Sandrin E, Gillet N. Validation of a French version of the Dutch Work Addiction Scale (DUWAS).

[65] Goldberg LR. An alternative “description of personality”: the big-five factor structure. In: Personality and personality disorders. 1st ed. London: Routledge; 2002. p. 34–47. 10.1037//0022-3514.59.6.1216 2283588

[66] Plaisant O, Courtois R, Réveillère C, Mendelsohn G, John OP. Validation par analyse factorielle du Big Five Inventory français (BFI-Fr). French. [English translation] Plaisant O, Courtois R, Réveillère C, Mendelsohn G, John OP. Validation of the French Big Five Inventory (BFI-Fr) through factor analysis: convergent analysis with the NEO-PI-R. Analyse convergente avec le NEO-PI-R. Elsev. 2010. p. 97–106. 10.1016/j.amp.2009.09.003.

[67] Jermann F, Billieux J, Larøi F, et al. Mindful Attention Awareness Scale (MAAS): psychometric properties of the French translation and exploration of its relations with emotion regulation strategies. Psychol Assess. 2009;21(4):506. 10.1037/a0017032. 19947785 10.1037/a0017032

[68] Lovibond PF, Lovibond SH. The structure of negative emotional states: comparison of the Depression Anxiety Stress Scales (DASS) with the Beck Depression and Anxiety Inventories. Behav Res Ther. 1995;33(3):335–43. 10.1016/0005-7967(94)00075-U. 7726811 10.1016/0005-7967(94)00075-u

[69] Martin D. [French translation of the Depression Anxiety Stress Scale (DASS-21), Ottawa University]. n.d. Available from: https://www2.psy.unsw.edu.au/groups/dass/French/French.htm.

[70] Zenasni, Untas, Nelson, Sonnentag, 2014. [French translation of the psychological detachment from work scale]. Personal communication. Accessed 12 Apr 2014.

[71] Team RC. R. A language and environment for statistical computing. Vienna: R Foundation for Statistical Computing; 2020.

[72] Rosseel Y. lavaan: an R package for structural equation modeling. J Stat Software. 2012;48:1–36. 10.18637/jss.v048.i02.

[73] Tucker LR, Lewis C. A reliability coefficient for maximum likelihood factor analysis. Psychometrika. 1973;38(1):1–10. 10.1007/BF02291170.

[74] Bentler PM. Comparative fit indexes in structural models. Psychol Bull. 1990;107(2): 238. 10.1037/0033-2909.107.2.238. 2320703 10.1037/0033-2909.107.2.238

[75] Cook KF, Kallen MA, Amtmann D. Having a fit: impact of number of items and distribution of data on traditional criteria for assessing IRT’s unidimensionality assumption. Qual Life Res. 2009;18:447–60. 10.1007/s11136-009-9464-4. 19294529 10.1007/s11136-009-9464-4 PMC2746381

[76] Bentler P. EQS structural equations program manual. Encino: Multivariate Software; 1995.

[77] Bentler PM, Bonett DG. Significance tests and goodness of fit in the analysis of covariance structures. Psychol Bull. 1980;88(3): 588. 10.1037/0033-2909.88.3.588.

[78] Browne MW, Cudeck R. Alternative ways of assessing model fit. Soc Methods Res. 1992;21(2):230–58. 10.1177/0049124192021002005.

[79] Hu Lt, Bentler PM. Cutoff criteria for fit indexes in covariance structure analysis: conventional criteria versus new alternatives. Struct Equ Modeling. 1999;6(1):1–55. 10.1080/10705519909540118.

[80] Widaman KF, Reise SP. Exploring the measurement invariance of psychological instruments: applications in the substance use domain. Am Psychol Assoc. 1997. 10.1037/10222-009.

[81] Chen FF. Sensitivity of goodness of fit indexes to lack of measurement invariance. Struct Equ Modeling. 2007;14(3):464–504. 10.1080/10705510701301834.

[82] Cronbach LJ. Coefficient alpha and the internal structure of tests. Psychometrika. 1951;16(3):297–334. 10.1007/BF02310555.

[83] McDonald RP. Test theory: a unified treatment. Psychol Press; 2013. 10.4324/9781410601087.

[84] Jorgensen TD, Pornprasertmanit S, Schoemann AM, Rosseel Y. SemTools: useful tools for structural equation modeling. R package version 05–6. Available from: https://cran.r-project.org/web/packages/semTools/semTools.pdf. Accessed 13 May 2024.

[85] Trizano-Hermosilla I, Alvarado JM. Best alternatives to Cronbach’s alpha reliability in realistic conditions: congeneric and asymmetrical measurements. Front Psychol. 2016;7:769. 10.3389/fpsyg.2016.00769. 27303333 10.3389/fpsyg.2016.00769 PMC4880791

[86] DeVellis RF, Thorpe CT. Scale development: theory and applications. Thousand Oaks: Sage Publications; 2021.

[87] Fornell C, Larcker DF. Evaluating structural equation models with unobservable variables and measurement error. J Mark Res. 1981;18(1):39–50. 10.1177/002224378101800104.

[88] Henseler J, Ringle CM, Sarstedt M. A new criterion for assessing discriminant validity in variance-based structural equation modeling. J Acad Mark Sci. 2015;43:115–35. 10.1007/s11747-014-0403-8.

[89] Stanley D. apaTables: create American Psychological Association (APA) style tables (2.0. 8)[R]. 2021.

[90] McMillan LH, O’Driscoll MP, Burke RJ. Workaholism: a review of theory, research, and future directions. Int Rev Ind Org Psychol. 2003;2003(18):167–89. 10.1002/0470013346.

[91] Aziz S, Bellows G, Wuensch K. The relationship between workaholism and negative affect: mindfulness matters! Int J Ment Health Addict. 2021;19:1605–14. 10.1007/s11469-020-00249-5.

[92] Howard GJ, Smith RW, Haynes NJ, Clark MA. Being mindful about workaholism: associations between dimensions of workaholism and mindfulness. Occup Health Sci. 2022;6(2):295–311. 10.1007/s41542-022-00113-z.

[93] Pflügner K, Maier C, Weitzel T. The direct and indirect influence of mindfulness on techno-stressors and job burnout: a quantitative study of white-collar workers. Comput Hum Behav. 2021;115: 106566. 10.1016/j.chb.2020.106566.

[94] Haun VC, Nübold A, Bauer AG. Being mindful at work and at home: buffering effects in the stressor–detachment model. J Occup Org Psychol. 2018;91(2):385–410. 10.1111/joop.12200.

[95] Hülsheger UR, Lang JW, Depenbrock F, Fehrmann C, Zijlstra FR, Alberts HJ. The power of presence: the role of mindfulness at work for daily levels and change trajectories of psychological detachment and sleep quality. J Appl Psychol. 2014;99(6):1113. 10.1037/a0037702. 25198098 10.1037/a0037702

[96] Jamieson SD, Tuckey MR. Mindfulness interventions in the workplace: a critique of the current state of the literature. J Occup Health Psychol. 2017;22(2):180. 10.1037/ocp0000048. 27643606 10.1037/ocp0000048

[97] Galla B, Baelen RN, Fiore HM, Hutt S, Shenhav A. Social media desire and impulsiveness: Intensified by self-immersion, reduced by mindfulness. University of Pittsburgh; 2018. 10.31234/osf.io/ch43n.

[98] Woodlief D, Taylor SG, Fuller M, Malone PS, Zarrett N. Smartphone use and mindfulness: empirical tests of a hypothesized connection. Mindful. 2024:1–17. 10.1007/s12671-024-02349-y.

[99] Reinecke L, Aufenanger S, Beutel ME, et al. Digital stress over the life span: the effects of communication load and internet multitasking on perceived stress and psychological health impairments in a German probability sample. Medi Psychol. 2017;20(1):90–115. 10.1080/15213269.2015.1121832.

[100] Misra S, Stokols D. Psychological and health outcomes of perceived information overload. Environ Behav. 2012;44(6):737–59. 10.1177/0013916511404408.

[101] Hefner D, Vorderer P. Digital stress: permanent connectedness and multitasking. In: Reinecke L, Oliver MB, editors. The Routledge handbook of media use and well-being. New York: Routledge; 2016. p. 237–49.

[102] Ordinance on human research with the exception of clinical trials. Available from: https://www.fedlex.admin.ch/eli/cc/2013/642/en.

