# Supplementary material for: Psychometric properties of telepressure measures in the workplace and private life among French-speaking employees
Source: BMC Psychol. 2025 Apr 3;13:329. doi: 10.1186/s40359-025-02616-0 (PMC11966854; doi:10.1186/s40359-025-02616-0)
Supplement: Supplementary file 1 — Supplementary Material 1. [file 40359_2025_2616_MOESM1_ESM.docx]

**Supplementary material of the paper “Psychometric Properties of Telepressure Measures in the Workplace and Private Life among French-Speaking Employees”**

**Table S1. Sample characteristics**

| **Variables** |  | **N (%)** | **M (SD)** |
| --- | --- | --- | --- |
| Gender |  |  |  |
|  | Female | 200 (57.6%) |  |
|  | Male | 146 (42.1%) |  |
|  | Other | 1 (0.3%) |  |
| Age (in years) |  |  | 36.8 (10.7) |
| Job tenure (in years) |  |  | 5.1 (5.6) |
| Weekly work hours |  |  | 39.1 (7.5)^1^ |
| Employment rate (in %) |  |  | 91.7 (14.6) |
| Management role |  |  |  |
|  | Yes | 137 (39.5%) |  |
|  | No | 210 (60.5%) |  |
| Marital status |  |  |  |
|  | Single | 199 (57.3%) |  |
|  | Married | 88 (25.4%) |  |
|  | Concubinage | 25 (7.2%) |  |
|  | Divorced | 35 (10.1%) |  |
|  | Widowed | 0 (0%) |  |
| Education level^2^ |  |  |  |

**Table S1 (continued).**

|  | No formal education | 1 (0.3%) |  |
| --- | --- | --- | --- |
|  | Primary education | 2 (0.6%) |  |
|  | High school | 28 (8.1%) |  |
|  | Vocational training | 54 (15.6%) |  |
|  | Higher education | 262 (75.5%) |  |

**Notes:** ^1^ 344 / 347 participants reported their weekly work hours. ^2^ In Switzerland, “no formal education” refers to individuals who have not completed the compulsory primary school level, “primary education” refers to the completion of primary school, which is the first stage of compulsory education, “high school” refers to the completion of secondary education, “vocational training” refers to the completion of vocational education and training, which combines classroom instruction with hands-on work experience, and “higher education” refers to the completion of tertiary education, which includes universities and higher professional education and training institutions.

**French version of the workplace telepressure measure**

**Consigne : Pour les questions suivantes, réfléchissez à la manière dont vous utilisez la technologie pour communiquer avec les personnes *de votre environnement professionnel*. Pensez plus particulièrement aux technologies basées sur *les messages numériques liés au travail* qui vous permettent de contrôler le moment où vous répondez (p. ex. courriel, messages texte, messagerie vocale). Veuillez indiquer dans quelle mesure vous êtes d'accord ou non avec les affirmations suivantes:**

**Choisissez la réponse appropriée pour chaque élément :**

|  | 1 - Pas du tout d'accord | 2 | 3 | 4 | 5 - Tout à fait d'accord |
| --- | --- | --- | --- | --- | --- |
| Il est difficile pour moi de me concentrer sur autre chose lorsque je reçois un message professionnel de quelqu'un. |  |  |  |  |  |
| Je peux mieux me concentrer sur d'autres tâches une fois que j'ai répondu à mes messages professionnels. |  |  |  |  |  |
| Je ne peux pas m'empêcher de penser à un message professionnel jusqu'à ce que j'y ai répondu. |  |  |  |  |  |
| Je ressens un fort besoin de répondre immédiatement aux autres. |  |  |  |  |  |
| J’ai un sentiment irrépressible de devoir répondre pile au moment où je reçois une demande professionnelle de quelqu’un. |  |  |  |  |  |
| Il est difficile pour moi de résister à l'envie de répondre tout de suite à un message professionnel. |  |  |  |  |  |

**French version of the private life telepressure measure**

**Consigne : Pour les questions suivantes, réfléchissez à la manière dont vous utilisez la technologie pour communiquer avec les personnes *de votre environnement personnel*. Pensez plus particulièrement aux technologies basées sur *les messages numériques liés à votre vie personnelle* qui vous permettent de contrôler le moment où vous répondez (p. ex. courriel, messages texte, messagerie vocale). Veuillez indiquer dans quelle mesure vous êtes d'accord ou non avec les affirmations suivantes :**

**Choisissez la réponse appropriée pour chaque élément :**

|  | 1 - Pas du tout d'accord | 2 | 3 | 4 | 5 - Tout à fait d'accord |
| --- | --- | --- | --- | --- | --- |
| Il est difficile pour moi de me concentrer sur autre chose lorsque je reçois un message personnel de quelqu'un. |  |  |  |  |  |
| Je peux mieux me concentrer sur d'autres tâches une fois que j'ai répondu à mes messages personnels. |  |  |  |  |  |
| Je ne peux pas m'empêcher de penser à un message personnel jusqu'à ce que j'y ai répondu. |  |  |  |  |  |
| Je ressens un fort besoin de répondre immédiatement aux autres. |  |  |  |  |  |
| J’ai un sentiment irrépressible de devoir répondre pile au moment où je reçois une demande personnelle de quelqu’un. |  |  |  |  |  |
| Il est difficile pour moi de résister à l'envie de répondre tout de suite à un message personnel. |  |  |  |  |  |

**English version of the workplace telepressure measure**

**Stem: For the following questions, think about how you use technology to communicate with people *in your workplace*. Specifically think about *work-related message-based technologies* that allow you to control when you respond (email, text messages, voicemail, etc.). Please rate how much you agree or disagree with the statements:**

**Choose the appropriate answer for each item:**

|  | 1 – Strongly disagree | 2 | 3 | 4 | 5 – Strongly agree |
| --- | --- | --- | --- | --- | --- |
| It is hard for me to focus on other things when I receive a work-related message from someone. |  |  |  |  |  |
| I can concentrate better on other tasks once I have responded to my work-related messages. |  |  |  |  |  |
| I cannot stop thinking about a work-related message until I have responded. |  |  |  |  |  |
| I feel a strong need to respond to others immediately. |  |  |  |  |  |
| I have an overwhelming feeling to respond right at that moment when I receive a work-related request from someone. |  |  |  |  |  |
| It is difficult for me to resist responding to a work-related message right away. |  |  |  |  |  |

**English version of the private life telepressure measure**

**Stem: For the following questions, think about how you use technology to communicate with people *in your private life*. Specifically think about *personal message-based technologies* that allow you to control when you respond (email, text messages, voicemail, etc.). Please rate how much you agree or disagree with the statements:**

**Choose the appropriate answer for each item:**

|  | 1 – Strongly disagree | 2 | 3 | 4 | 5 – Strongly agree |
| --- | --- | --- | --- | --- | --- |
| It is hard for me to focus on other things when I receive a personal message from someone. |  |  |  |  |  |
| I can concentrate better on other tasks once I have responded to my personal messages. |  |  |  |  |  |
| I cannot stop thinking about a personal message until I have responded. |  |  |  |  |  |
| I feel a strong need to respond to others immediately. |  |  |  |  |  |
| I have an overwhelming feeling to respond right at that moment when I receive a personal request from someone. |  |  |  |  |  |
| It is difficult for me to resist responding to a personal message right away. |  |  |  |  |  |
